# Node embedding-based graph autoencoder outlier detection for adverse pregnancy outcomes

**DOI:** 10.1038/s41598-023-46726-4

**Published:** 2023-11-14

**Authors:** Wasif Khan, Nazar Zaki, Amir Ahmad, Mohammad M. Masud, Romana Govender, Natalia Rojas-Perilla, Luqman Ali, Nadirah Ghenimi, Luai A. Ahmed

**Affiliations:** 1 Department of Computer Science and Software Engineering, College of Information Technology, United Arab Emirates University, P.O. Box 15551, Al Ain, United Arab Emirates; 2 ASPIRE Precision Medicine Research Institute Abu Dhabi (ASPIREPMRIAD), Al Ain, United Arab Emirates; 3 Department of Information Systems and Security, College of Information Technology, United Arab Emirates University, P.O. Box 15551, Al Ain, United Arab Emirates; 4 Department of Family Medicine, College of Medicine and Health Sciences, United Arab Emirates University, P.O. Box 15551, Al Ain, United Arab Emirates; 5 Department of Analytics in the Digital Era, United Arab Emirates University, P.O. Box 15551, Al Ain, United Arab Emirates; 6 Institute of Public Health, College of Medicine and Health Sciences, United Arab Emirates University, P.O. Box 15551, Al Ain, United Arab Emirates; 7 Zayed Centre for Health Sciences, United Arab Emirates University, P.O. Box 15551, Al Ain, United Arab Emirates

**Keywords:** Biomedical engineering, Preterm birth, Data mining

## Abstract

Adverse pregnancy outcomes, such as low birth weight (LBW) and preterm birth (PTB), can have serious consequences for both the mother and infant. Early prediction of such outcomes is important for their prevention. Previous studies using traditional machine learning (ML) models for predicting PTB and LBW have encountered two important limitations: extreme class imbalance in medical datasets and the inability to account for complex relational structures between entities. To address these limitations, we propose a node embedding-based graph outlier detection algorithm to predict adverse pregnancy outcomes. We developed a knowledge graph using a well-curated representative dataset of the Emirati population and two node embedding algorithms. The graph autoencoder (GAE) was trained by applying a combination of original risk factors and node embedding features. Samples that were difficult to reconstruct at the output of GAE were identified as outliers considered representing PTB and LBW samples. Our experiments using LBW, PTB, and very PTB datasets demonstrated that incorporating node embedding considerably improved performance, achieving a 12% higher AUC-ROC compared to traditional GAE. Our study demonstrates the effectiveness of node embedding and graph outlier detection in improving the prediction performance of adverse pregnancy outcomes in well-curated population datasets.

## Introduction

More than 254 million pregnancies were recorded each year between 2015 and 2019^1^, of which > 73 million ended in abortion^2^. Consequently, ˃140 million babies are born annually around the world^3^. In Asia, there are 16 live births per 1000 people; the corresponding figure in the United Arab Emirates (UAE) is 10 live births per 1000 people^4^ Some infants are born preterm (PTB) and/or with low birthweight (LBW). Newborns weighing < 2500 g are considered to be LBW infants, which can be caused by several factors, such as high parity, maternal diet, shorter interpregnancy intervals, premature delivery, and socioeconomic issues. PTB and very PTB (vPTB) refer to the delivery of an infant before 37 and 32 weeks of gestation, respectively. PTB and LBW are strongly associated with each other^5–12^. For example, a PTB infant is 18 times more at risk of being an LBW infant and vice versa^12^. Furthermore, LBW and PTB have substantial health impacts^11^ and are important determinants of infant health and survival. Compared to infants with normal birth weight, LBW infants are at a higher risk of perinatal death and have a greater chance of developing serious developmental problems, including mental retardation, low IQ, visual and auditory impairments, long-term disabilities, and premature death^13–15^. In contrast, PTB can place the child at a higher risk of serious health issues, such as gastrointestinal, respiratory, hearing, vision, cognitive, and growth problems as well as correlating a greater chance of permanent disability and death^16^. PTB is a major global concern that affects > 15 million infants every year, of which almost 1 million do not survive^17^. To increase awareness and improve treatment, World Prematurity Day and Prematurity Awareness Month are observed on November 17 and every November, respectively^18,19^. With a prevalence of 6.3%, PTB is a concern in the UAE^12^. Efforts have been made to prevent PTB births; however, their prevalence is still high^20^. Early detection and management can improve outcomes for both mothers and infants. In recent years, machine learning (ML) models have shown promising performance in various domains, such as in the field of obstetrics for LBW and PTB prediction.

Although many studies for LBW and PTB prediction have been performed (Table 1) these efforts have two major limitations. First, many pregnancies occur each year; however, the medical datasets are not publicly available due to privacy reasons, and most of the datasets often suffer from class imbalance, making it difficult to accurately predict minority classes. Data balancing techniques can be employed; however, they do not always effectively capture the distribution of the minority class, resulting in subpar performance. Anomaly detection algorithms can help address this issue. Second, ML models overlook inter-entity relationships and rely only on grid-based data^18,19^. As a result, patients in a dataset are treated as independent and uncorrelated^21^. This is not always accurate because patients can be correlated based on shared diseases or comorbidities^12,20^. These complex relational structures pose a challenge to the ability of ML models to extract meaningful information from data. Consequently, the effectiveness of ML models in uncovering valuable insights is hindered.

**Table 1:** Previous studies on the outcomes of adverse pregnancies.

Ref	Year	Risk factor selected with justification	Feature engineering	Knowledge graph-based modeling
Feng et al.^23^	2019			
Kuhle et al.^32^	2018			
Sebthilkumar et al.^49^	2015			
Borson et al.^34^	2020			
Loreto et al.^35^	2019		✓	
Kumar et al.^50^	2020			
Anisha et al.^51^	2017		✓	
Faruk et al.^52^	2018			
Akhtar et al.^53^	2020			
Akhtar et al.^54^	2019			
Al Habashneh et al.^55^	2012			
Li et al.^15^	2020		✓	
Desiani et al.^13^	2019			
Ahmadi et al.^56^	2017			
Hussain et al.^57^	2020		✓	
Lu et al.^58^	2019			
Akbulut et al.^59^	2018			
Trujillo et al.^24^	2020			
Mercer et al.^27^	1996		✓	
Lee et al.^28^	2019		✓	
Tran et al.^29^	2016		✓	
Taha et al.^12^	2020			
Sun et al.^30^	2022		✓	
Koivu et al.^31^	2020		✓	
Raja et al.^60^	2021		✓	
Belaghi et al.^33^	2021	✓	✓	
Belagi et al.^36^	2021	✓	✓	
Diaz et al.^37^	2021		✓	
Lee et al.^38^	2021		✓	

To overcome these limitations, we propose a novel method to predict adverse pregnancy outcomes (LBW, PTB, and vPTB) using a node embedding-based graph outlier detection algorithm^22^. The knowledge graph is developed from a well-curated dataset representative of the Emirati population, and a GAE is employed for outlier detection. Our solution considerably improves the performance of the LBW and PTB prediction models in both parous and nulliparous women, demonstrating the effectiveness of node embedding and graph outlier detection.

## Related works

In this section, we provide a brief overview of studies conducted on adverse pregnancy outcomes using ML-based models. For example, Feng et al.^23^ utilized ultrasound features to predict fetal weight using data from 7875 women with 190 LBW samples. Trujillo et al.^24^ used support vector regression with a radial basis function kernel to estimate BW from a dataset of 250 women and 23 features such as maternal height, weight, and body mass index (BMI).

Similarly, various ML models (such as RF, SVM, NB, LR, DT, KNN, neural network, MLP, and ensemble models) have been used in various studies to predict LBW in infants^6^. The features used in these studies include average clinical attachment loss, clinical measures, education, gender, gestation age, height, hypertension condition, income, last weight recorded before conceiving, maternal age, medical history, mother’s age, newborn weight, number of children, parents’ education, periodontal parameters, place of residence, prepregnancy BMI, weight gain during pregnancy, and smoking. These studies used datasets ranging from 189 to 215,568 patients and achieved accuracy ranging from 72 to 97% with varying specificity and sensitivity. Important risk factors associated with LBW included the last weight recorded before conceiving, mother’s age, prepregnancy BMI, and weight gain during pregnancy. Further details can be found in^25,26^.

There is a comprehensive literature on PTB analysis from the medical perspective; however, there are limited studies predicting PTB using ML algorithms. For instance, Mercer et al.^27^ performed logistic regression-based analysis for PTB infants using data from 2929 women (multiparous: 1711, nulliparous: 1218). Lee et al.^28^ proposed ML methods to predict PTB and its important risk factors on a dataset of 596 patients. Further, they revealed that BMI was the most important risk factor followed by hypertension, diabetes mellitus, prior cone biopsy, and prior placenta previa for PTB prediction. Tran et al.^29^ proposed stabilized sparse logistic regression for PTB prediction and estimated important risk factors on a dataset of 15,814 women for PTB prediction, achieving an AUC of 0.85 at 34 weeks of gestation.

Taha et al.^12^ conducted a study based on statistical analysis to identify the factors associated with PTB and LBW in the UAE. They used a dataset of 1610 (Emiratis and expatriates) mothers from Abu Dhabi, UAE. Furthermore, they showed that PTB and LBW are highly associated with each other. They also revealed that Arab national women are twice at a higher risk of having PTB compared to non-Arab mothers. Sun et al.^30^ evaluated multiple ML algorithms and found that RF performed well (AUC of 0.89), with age, waist size, height, mean platelet volume, globulins, and serum inorganic phosphorus being key risk factors associated with PTB.

Koivu et al.^31^ used the CDC and NYC datasets (for external validation). An AUC of 0.67 was achieved for the CDC dataset, while a maximum AUC of 0.64 was achieved for the NYC dataset using the ANN and LightGBM models. Raja et al.^32^ also employed an ML model for PTB prediction. Belaghi et al.^33^ created a dataset of 112,963 nulliparous women with various risk factors selected based on the literature^34,35^ for PTB prediction. Belaghi et al.^36^ performed multivariate statistical analysis on a dataset of 267,226 births for PTB and spontaneous PTB in multiparous and nulliparous women. They found that the AUC using logistic regression in the first trimester was 0.68 and 0.73 for nulliparous and multiparous women, respectively, while it was 0.72 and 0.78, respectively, in the second trimester.

Diaz et al.^37^ proposed a ML model-based methodology for PTB prediction in chronodisrupted mothers. They used a dataset of 380 births (preterm: 157, term: 223) and showed that features, such as sleeping habits, were important for PTB prediction. Lee et al.^38^ used multiple ML models (LR, ANN, RF) for PTB using 90 different features, including particulate matter and depression. They used a dataset of 405,586 participants for the classification of PTB into four different categories. However, the dataset was highly imbalanced (2.22% in the minority class). Experiments were performed to show that an AUC of 0.52–0.58 was achieved.

## Methods

**Figure 1:**
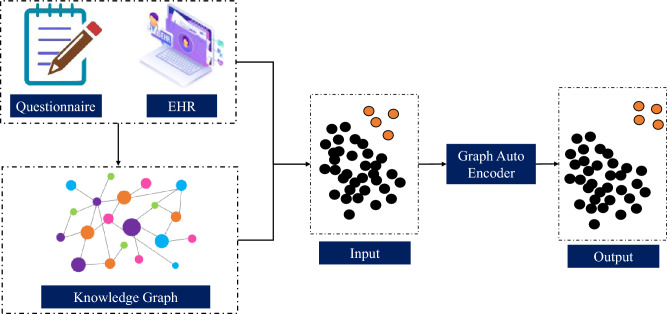
Proposed methodology for a node embedding-based graph outlier detector.

An overview of the proposed system, which contains several modules, is shown in Fig. 1. In the first module, tabular data are transformed into a knowledge graph, and node embedding features are extracted. Node embedding vectors and tabular data are fed as inputs into the graph auto encoder (GAE). The yellow dots in Fig. 1 at the input of the GAE signify outliers, which are challenging to reconstruct at the output of the GAE because of their deviation from the norm. This deviation results in an increased reconstruction error; therefore, these points are referred to as outliers. We evaluate the results based on different metrics such as AUC and AUC-PR. Each module is explained in detail below.

### Ethics statement

Our analysis used data from a prospective cohort study conducted in Al Ain, Abu Dhabi, UAE. The study included pregnant women from the Emirati population who were recruited and followed up via medical records in hospitals. As described in a previous study^39^, the participants completed a baseline questionnaire at recruitment. The study received approval from the Abu Dhabi Health Research and Technology Ethics Committee (DOH/CVDC/2022/72) and was in complete agreement with the Declaration of Helsinki. All participants provided written informed consent prior to data collection. Furthermore, all experiments were performed in accordance with relevant guidelines and regulations.

### Dataset

The dataset used in this analysis was previously described and was obtained from an ongoing prospective maternal and child cohort, the Mutaba`ah Study, in Al Ain, UAE^39^. The risk factors used in this analysis were selected based on the literature and medical justification. Parous is defined as having given birth to a child, and nulliparous denotes a woman who has not given birth to a child. Data for 3508 patients were used, of which 2708 were parous (2411 with normal births and 297 with PTB) and 801 were nulliparous (of which 97 had experienced PTB). Only 35 of the 2708 parous women had experienced vPTB, whereas 22 of the 801 nulliparous women had vPTB delivery. Full details are provided in Table 2.

**Table 2:** Distribution of the dataset.

Dataset name	Parity	Total samples	Normal samples	Abnormal samples	Abnormality (%)
LBW	Parous	2668	2407	261	11.1
LBW	Nulliparous	785	655	130	16.5
PTB	Parous	2708	2411	297	10.9
PTB	Nulliparous	801	704	97	12.1
Very PTB	Parous	2708	2653	35	1.3
Very PTB	Nulliparous	801	779	22	2.7

### Problem formulation and graph construction

An outlier, or anomaly, refers to instances in a dataset that considerably deviate from the majority of observations^40^. Despite their rarity, outliers can provide valuable insights and information. In our particular dataset, for example, vPTB samples constitute only a small fraction (1.3%) of the total observations, but their detection and prediction are important. Our method builds upon a dataset $D$ that comprises patients with varying risk factors. Based on GAE, reconstructing patients who are rare (PTB, vPTB) is challenging, leading to higher reconstruction errors; as such, they are considered to be outliers. Our approach was inspired by Du et al.^22^; however, we introduced a new graph construction method. Moreover, our approach utilizes node embedding rather than a cosine similarity adjacency matrix, leading to improved GAE predictive capabilities. To construct a graph, various entities, such as patients, demographics, pregnancy conditions, medical history before pregnancy, and fetal characteristics, were identified as nodes. Each patient was assigned a unique identifier to create a distinct node, and relationships were established between the nodes to show edges. The topology of the graph was determined based on the similarity of its node properties such that patients with similar complications were positioned closer together^25,41^. To implement this in Neo4j, we used primary key and foreign key concepts to create nodes and relationships between them. In particular, we created a node for each entity identified in the text, such as a patient node, demographics node, and pregnancy condition node. Each node exhibits its own set of properties such as patient age, medical history, and fetal characteristics. The relationships between nodes were established based on the relationships identified in the text. For example, we created a "HAS_DEMOGRAPHICS" relationship between a patient node and a demographic node to indicate that the patient has demographic information.

### Node2Vec

The Node2Vec node embedding algorithm was employed to capture the structural and semantic relationships between nodes and learn their low-dimensional representations. Node2vec is a scalable algorithm that employs a flexible objective function balancing the preservation of local and global graph structures. Based on a graph G = (V, E), Node2Vec learns an embedding for each node in the graph such that the embeddings capture the structural information of the graph. It generates random walks from the graph, which form sequences of nodes obtained by randomly traversing the graph, with a biased transition probability that balances between breadth-first and depth-first search strategies. For each node in the graph, the algorithm samples a set of random walks by performing a fixed number of steps, where each step follows a transition probability based on the node's proximity to the current node, as controlled by two hyperparameters, $p$ and $q$. The transition probability is then defined as follows:

$${P}_{xy}=\left\{\begin{array}{c}\frac{1}{p} \quad if\, {d}_{xy}=0\\ 1 \quad if\, {d}_{xy}=1 \\ \frac{1}{q} \quad otherwise\end{array}\right.$$

where ${d}_{xy}$ is the shortest path distance between nodes $x$ and $y$; $p$ and $q$ control the possibility of returning to the previous node and exploring new nodes, respectively. After generating random walks, the Node2Vec algorithm optimizes a skip-gram model to learn node embeddings that capture the local and global structural information of the graph. In particular, the skip-gram model aims to maximize the possibility of predicting context nodes given the target node in the random walks. Details regarding the Node2Vec algorithm can be found in^25^ and^42^.

### FastRP

FastRP is a rapid and scalable algorithm for learning low-dimensional representations of nodes in large-scale graphs^43^. FastRP is based on randomized projections that map high-dimensional vectors to lower-dimensional space while preserving pairwise distances. This approach allows us to compute low-dimensional vector representations for each node in a graph, which can then be used for various downstream tasks. The FastRP algorithm factorizes the adjacency matrix of a graph into two low-rank matrices, representing row and column embeddings. Row embeddings are computed using a randomized projection technique that involves multiplying the adjacency matrix with a random Gaussian matrix. This results in a low-rank approximation of the adjacency matrix that preserves the pairwise distances between nodes. Column embeddings are obtained by transposing the row embeddings. The FastRP algorithm is defined as $A \approx XWXT$, where $A$ is the adjacency matrix of the graph, $X$ is the row embeddings, $W$ is the projection matrix, and $XT$ is the column embeddings. The projection matrix $W$ is computed by multiplying $A$ by a random Gaussian matrix $R$, then orthogonalizing the resulting matrix using $QR$ decomposition. Row embeddings $X$ are obtained by multiplying $A$ by projection matrix $W$, whereas column embeddings $XT$ are obtained by transposing $X$.

### Graph auto encoder

GAE provides a powerful unsupervised learning framework for obtaining meaningful representations of graph-structured data. It comprises an encoder, a decoder, and a loss function that work together to learn the features between nodes and their neighbors. The encoder maps input data into a lower-dimensional representation, while the decoder maps the representation back to the data. For dataset $X$, its associated node embedding from graph $G$, weight matrices ${W}^{\left(i\right)}$, and bias matrices ${b}^{\left(i\right)}$, the GAE can be represented as follows:

$$Z=f\left(X,A\right)=LeakyReLU((LeakyReLU(X\cdot G\cdot {W}^{\left(0\right)}-{b}^{\left(0\right)})G\cdot {W}^{\left(1\right)}-{b}^{\left(1\right)})$$

The loss function, which measures the difference between the reconstructed output of GAE $Z$ and the input ${X}^{\prime}$, is as follows:

$$f\left(X,A\right)=\sum \left(L\left({X}^{\prime},Z\right)\right)=\sum {\Vert {X}^{\prime}-Z\Vert }^{2}$$

The aim of GAE training is to uncover the underlying relationships between any patient ${p}_{i}$ and its neighboring patients. At the output of the GAE, ${p}_{i}$ and its neighbors are reconstructed such that the reconstruction error is minimized. Because the majority of the patients experience normal circumstances, they will be easier to reconstruct through GAE. Outliers present a challenge in the reconstruction because they significantly diverge from the norm.

### Evaluation metrics

We used two commonly used performance metrics, namely, the area under the receiver operating characteristic (ROC) curve and the area under the precision–recall (PR) curve (AUC-PR), to evaluate the performance of the proposed method^44^. AUC-ROC and AUC-PR are important performance metrics for evaluating ML models. AUC-ROC measures the ability of the classifier to distinguish between positive and negative classes, whereas AUC-PR measures the precision–recall trade-off of the classifier. Details regarding the calculation and choice of these metrics can be found in^25^ and^44^.

## Experiments and results

**Figure 2:**
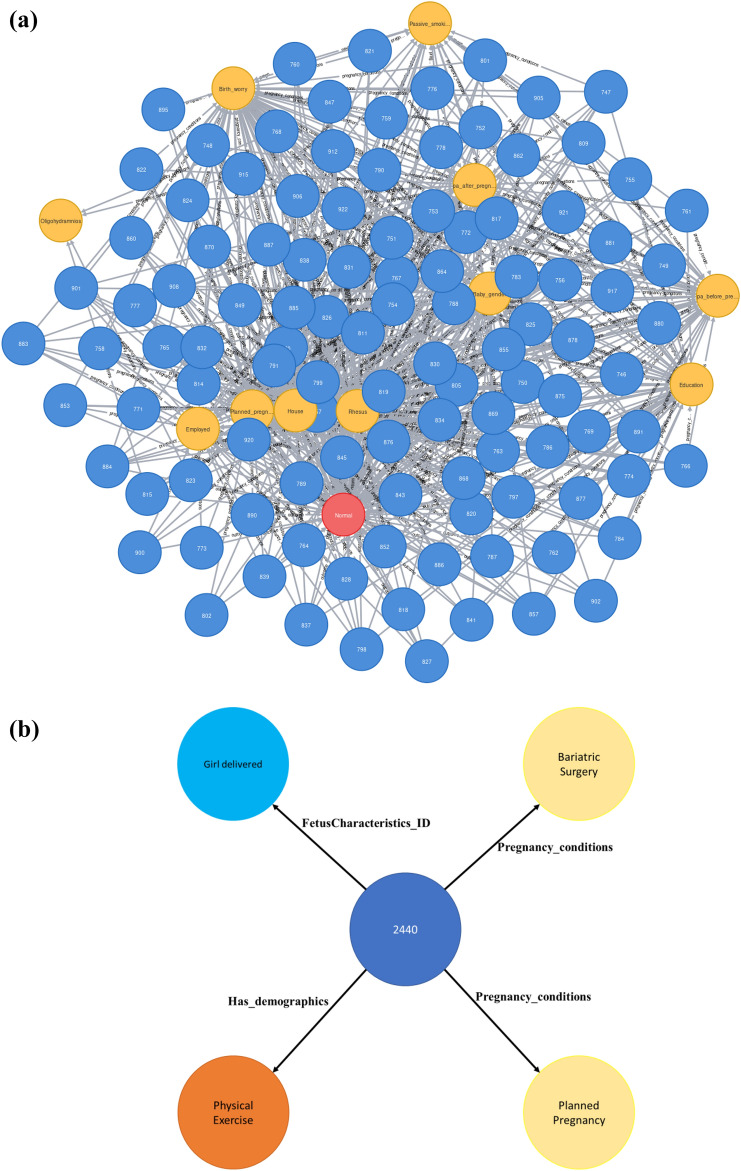
(**a**) Knowledge graph sample. Blue, yellow, and red nodes represent patients with their respective PIDs, risk factors, and target class information, respectively. (**b**) Sample from the congested graph for clear demonstration.

Graph construction was performed using Neo4j, and GAE was performed using MATLAB 2018B. All experiments were repeated ten times and the average results are presented. After performing knowledge graph creation, we were able to identify 2737 and 830 entities, known as nodes, by establishing 19,987 and 6061 relationships for parous and nulliparous patients, respectively. These include 2698 and 815 nodes with 19,203 and 5812 relationships for the LBW datasets in parous and nulliparous women, respectively. The graph produced from the initial risk factors using Neo4j is shown in Fig. 2.

**Figure 3:**
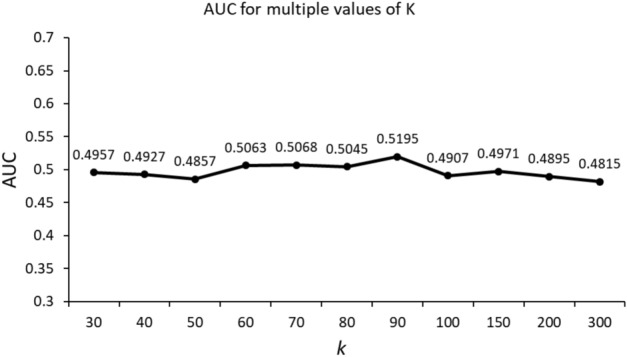
AUC for different values of k for GAE.

We adopted the same experimental setup as discussed in^22^ and initialized key hyperparameters, such as the learning rate and the architecture of hidden layers. We tried different learning rates and hidden layer structures to determine the best setup. Finally, we found that a learning rate of ${1e}^{-9}$ and a three-layer architecture worked best in our experiments. Traditional GAE is sensitive to the value of $k$; hence, we performed multiple experiments to determine the optimal value of k. The best performance was achieved for k = 90 (Fig. 3). Therefore, for GAE, the value of k was set to 90 for all experiments.

The performance results in Table 3 compare different methods for LBW prediction in parous and nulliparous women. The methods evaluated include original GAE; FastRP with 16, 32, and 64 features; Node2Vec with 16, 32, and 64 features; and a combination of FastRP and Node2Vec with features. The results show that the LBW prediction performance varies across different methods and feature sizes. The combination of FastRP and Node2Vec with 32 features performed relatively better than the other methods for LBW prediction in parous women with AUC-ROC and AUC-PR values of 0.6077 and 0.1545, whereas FastRP with 64 features achieved the best performance for nulliparous women with AUC-ROC and AUC-PR values of 0.5796 and 0.2305, respectively. The original GAE performed worst for parous and nulliparous women with AUC values of 0.4982 and 0.4891, respectively. The overall improvements in AUC-ROC for parous and nulliparous women using node embedding GAE were ~ 11% and 9%, respectively.

**Table 3:** Experimental results for LBW prediction using anomaly detection algorithms.

LBW prediction	Parous		Nulliparous	
Method	AUC	AUC-PR	AUC	AUC-PR
Original GAE	0.4982	0.0966	0.4891	0.1584
FastRP 16	0.5780	0.1280	0.5116	0.2434
FastRP 32	0.5773	0.1349	**0.5796**	**0.2305**
FastRP 64	0.4876	0.1002	0.5248	0.1970
Node2Vec 16	0.5311	0.1522	0.5116	0.2389
Node2Vec 32	0.5506	0.1541	0.5017	0.1722
Node2Vec 64	0.5268	0.1128	0.5244	0.1945
combine 16	0.5654	0.1439	0.5092	0.2058
combine 32	**0.6077**	**0.1545**	0.5428	0.1877
combine 64	0.5058	0.1086	0.5384	0.2022

Significant values are in bold.

The results of the PTB prediction in parous and nulliparous women are shown in Table 4. The AUC values for all methods are ~ 0.5. Among the methods for parous women, FastRP with 16 features performs the best. The AUC-ROC of the combined 64 features was higher; however, FastRP with 16 features achieved better performance with AUC-ROC and AUC-PR values of 0.5026 and 0.1491. For nulliparous women, the best AUC-ROC of 0.5647 was achieved using FastRP with 64 features that exhibits more than a 5% improvement relative to GAE-based predictions.

**Table 4:** Experimental results for PTB prediction using anomaly detection algorithms.

PTB prediction	Parous		Nulliparous	
Method	AUC	AUC-PR	AUC	AUC-PR
Original	0.5016	0.1208	0.5194	0.1337
FastRP 16	0.5026	0.1491	0.5070	0.1541
FastRP 32	0.5011	0.1168	0.5133	0.1604
FastRP 64	0.4945	0.0856	0.5647	0.1385
Node2Vec 16	0.5026	0.1490	0.5364	0.1550
Node2Vec 32	0.5011	0.1170	0.5129	0.1537
Node2Vec 64	0.4946	0.0863	0.5058	0.1270
combine 16	0.4990	0.0867	0.5124	0.1517
combine 32	0.4944	0.0854	0.5074	0.1292
combine 64	0.5078	0.1258	0.5287	0.1382

Table 5 shows the results for vPTB prediction. The best results for parous women were achieved using Node2Vec with 16 features, showing an AUC-ROC of 0.5756 with more than 7% improvement compared to the original GAE-based implementation. Similarly, an improvement of > 11% was observed for nulliparous women, which achieved an AUC-ROC of 0.6696 compared to 0.5555 achieved by the original GAE-based approach.

**Table 5:** Experimental results for vPTB prediction using anomaly detection algorithms.

vPTB prediction	Parous	Nulliparous		
Method	AUC	AUC-PR	AUC	AUC-PR
Original	0.4994	0.0145	0.5555	0.0379
FastRP 16	0.5426	0.0160	0.4904	0.0137
FastRP 32	0.5086	0.0221	0.6596	0.0477
FastRP 64	0.5029	0.0151	0.5041	0.0284
Node2Vec 16	**0.5756**	0.0251	0.5175	0.0332
Node2Vec 32	0.5087	0.0226	0.6155	0.0364
Node2Vec 64	0.5245	0.0141	**0.6579**	**0.0774**
combine 16	0.5596	0.0261	0.5263	0.0451
combine 32	0.5027	0.0143	**0.6696**	0.0491
combine 64	0.5045	0.0140	0.5298	0.0321

Significant values are in bold.

### Patient explanations

**Figure 4:**
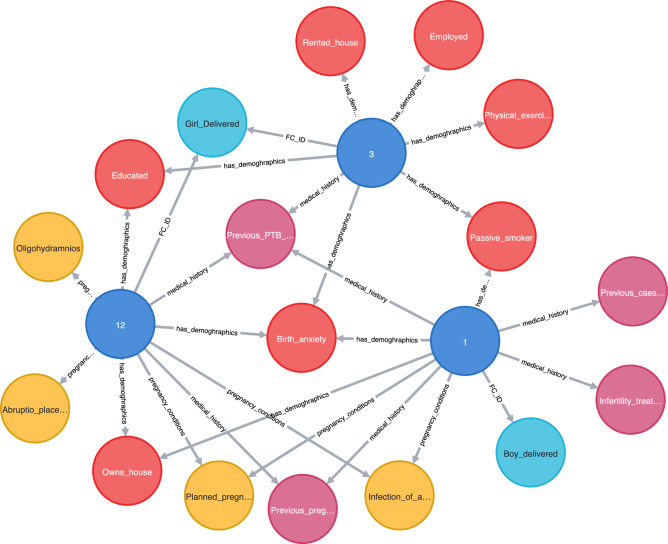
Explanation of GAE detection for PTB prediction.

Knowledge graphs offer a more effective approach to explain the reasons behind an outcome. Figure 4 shows three patients, PID 1, PID 3, and PID 12, who were predicted as outliers for PTB using the proposed method. Note that PID 12 did not experience PTB, although it was classified as an outlier using the GAE-based method. All three patients exhibited common risk factors, including anxiety about their upcoming birth and a prior history of PTB. Moreover, PID 12 shares additional risk factors with either PID 1 or PID 3, including previous pregnancy loss, infection of the amniotic sac, planned pregnancy, education level, and delivery of a female baby. In addition, the GAE-based method displayed the ability to identify specific risk factors for individual patients.

**Figure 5:**
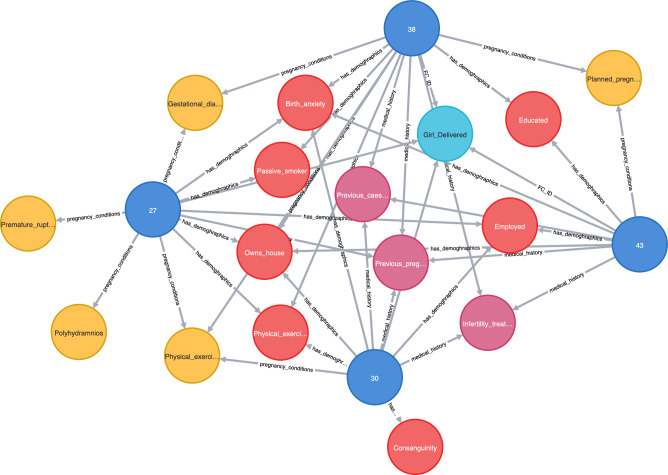
Predicted very PTB outlier patients using the GAE-based method.

**Figure 6:**
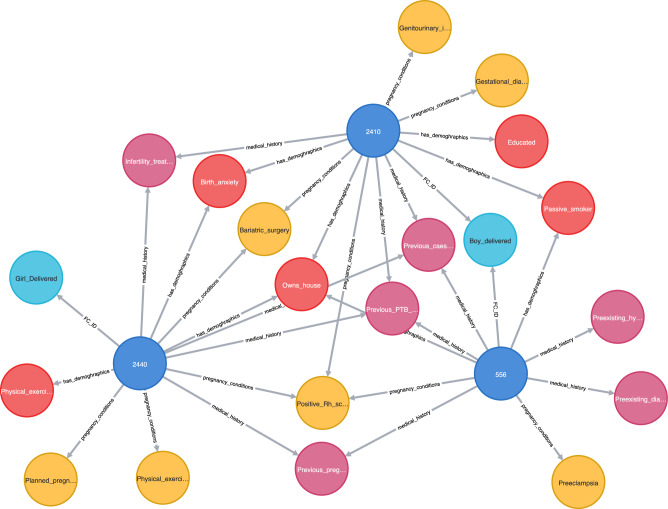
Predicted LBW outlier patients using the GAE-based method.

Figure 5 shows an example of patients with vPTB complication predicted as an outlier using the proposed method. The green patient nodes with IDs 43 and 30 are correctly detected as outliers, whereas patients 27 and 38, who did not experience vPTB, are detected as outliers by the GAE-based method. Figure 6 shows a use case for LBW, where two patient nodes (556 and 2410) are accurately recognized as outliers. However, the third patient node (PID 2440), who did not experience LBW, was wrongly classified as an outlier.

## Discussion

Herein, we used node embedding-based GAE outlier detection to address the limitations of traditional ML models in handling class imbalance and inter-entity relationships in medical datasets. This method detects outliers in graph-structured data, where each node and edge represent an individual patient and the relationships or interactions between patients, respectively. Notably, the prediction model performance was improved for PTB, vPTB, and LBW when we incorporated node embedding and GAE. For nulliparous women, an AUC-ROC of 0.6696 demonstrated an improvement of > 11% compared to an AUC-ROC of 0.5555 achieved using the original GAE-based approach. For parous women, an AUC-ROC of 0.5756 showed more than 7% improvement compared to the original GAE-based implementation. For LBW prediction, the AUC-ROC and AUC-PR values for parous women using node embedding GAE were 0.6077 and 0.1545, respectively, demonstrating an 11% improvement. Furthermore, the AUC-ROC and AUC-PR values for nulliparous women were 0.5796 and 0.2305, respectively, demonstrating a 7% improvement.

In addition to the improved predictions, we performed individual patient analysis for outlier (PTB and LBW) prediction. Based on our findings, we identified three patients (PID 1, PID 3, and PID 12) as outliers for PTB, indicating that all three patients are at higher risk of PTB. Notably, PID 12 was known to have delivered a full-term baby, despite sharing several risk factors with PID 1 and PID 3, such as infection of the amniotic sac, exposure to passive smoking, premature rupture of membranes, and concern for birth. These risk factors have been previously reported to be highly associated with PTB delivery^8,45–48^. There are several potential explanations for this anomaly. First, it should be noted that no predictive model, including autoencoder models, is 100% accurate. The performance of these models heavily relies on the quality and quantity of data used for training, and our model was trained on a relatively small dataset. Second, PTB can have multiple underlying causes and risk factors, some of which may not have been included in our list of risk factors used to train our model. Factors such as ultrasound parameters, biomarkers, and fetal fibronectin are known to contribute to PTB risk and may not have been captured in our dataset. Third, there may be human errors associated with the data used in this study during the data collection process or in the patient’s medical history. For example, the patient’s gestational age or delivery date may have been recorded incorrectly, or there may have been missing data that could have influenced the model’s predictions. These protective factors are related to the doctor–patient relationship. For example, receiving regular prenatal care from a healthcare provider can help identify and address any potential health issues that may increase the risk of PTB. Early intervention and optimal treatment of potential health issues such as hypertension and gestational diabetes, maintaining healthy diets and exercise, and regular cervical length assessments are some ways to help reduce the risk of PTB and promote the health and well-being of both the mother and baby. Similar explanations can be used to describe LBW anomalies.

Our model’s prediction for PTB and LBW showed improved performance compared with the traditional GAE approach; however, this result should always be considered in the context of the patient’s individual circumstances, medical history, and healthcare interventions provided. Moreover, the model can assist in the early detection of patients at risk of PTB and LBW, thus allowing for timely and personalized interventions to prevent adverse outcomes. These results can serve as a starting point for further research and pave the way for the development of more effective and accurate prediction models for adverse pregnancy outcomes.

PTB and LBW are the major causes of infant morbidity and mortality, and early prediction can help clinicians manage and prevent PTB and LBW. Our model was trained on a prenatal and clinical dataset to detect patterns associated with PTB/LBW. Consequently, our model can be used to predict the likelihood of PTB/LBW in new patients. Thus, clinicians can use this model to screen and risk stratify new patients as well as identify patterns in PTB/LBW data that are unique to individual patients, ultimately personalizing treatment plans that are tailored to the specific needs of each patient.

Some limitations to our model’s predictions must also be acknowledged. First, the dataset size and population diversity may not be representative of other populations. As such, larger and more diverse datasets may lead to different results, necessitating further investigation. However, the results of this study may be generalizable to other populations with appropriate modifications. The data used contain a majority of binary variables. Therefore, it is challenging to accurately capture and model inter-entity relationships. The proposed solutions may not effectively capture all complex relationships in the data, leading to less accurate performance. Furthermore, formal hyperparameter optimization procedures were not conducted. Instead, various settings were explored to assess their influence on the model’s performance, leaving room for potential improvements using advanced transformer-based generative models. Moreover, using a GAE and node embedding can be computationally expensive and may require high computational resources.

While our model’s prediction for PTB and LBW showed an improved performance compared with the traditional GAE approach, it is important to emphasize that the purpose of this research is to assist and augment the capabilities of clinicians rather than replace them. In addition, the practical implementation of this setup has not yet been adopted, presenting a potential avenue for future exploration. Furthermore, to ensure responsible and beneficial deployment of these technologies, it is important to consider the ethical considerations associated with the application of ML in healthcare, addressing concerns related to privacy, informed consent, and bias. Future studies could explore these issues in more detail and guide how to address them in practice.

## Conclusion

The results of this study demonstrate the potential for incorporating node embedding and graph outlier detection as a means of improving the accuracy of prediction models for adverse pregnancy outcomes (e.g., PTB and LBW), offering a unique advantage over traditional ML models. This prediction model can be a useful tool for identifying new patients at higher risk of PTB/LBW. By flagging these patients for further evaluation and interventions, this technique can potentially improve PTB/LBW outcomes and reduce associated health risks. Using the benefits of our predictive model, healthcare professionals can be proactive in PTB/LBW risk prediction and provide personalized and targeted patient-centered medical treatment, thus improving the morbidity and mortality outcomes in pregnancy.

## Data Availability

The data presented in this study can be made available on request from the Mutaba’ah Study. Approval from a research ethics committee may be required. Please contact Luai Ahmed (luai.ahmed.uaeu.ac.ae), the author responsible for data requests.

## References

[1] International Pregnancy | Guttmacher Institute. Accessed 24 May 2022. [Online]. Available: https://www.guttmacher.org/global/pregnancy

[2] Bearak J (2020). Unintended pregnancy and abortion by income, region, and the legal status of abortion: Estimates from a comprehensive model for 1990–2019. Lancet Glob. Health.

[3] Number of births per year. Accessed 24 May 2022. [Online]. Available: https://www.theworldcounts.com/populations/world/births

[4] Special Focus on Global Fertility WORLD POPULATION GLOBAL TOTAL FERTILITY RATE % OF ALL BIRTHS GLOBALLY TO MOTHERS AGES 35+.

[5] Teitelman AM, Welch LS, Hellenbrand KG, Bracken MB (1990). Effect of maternal work activity on preterm birth and low birth weight. Am. J. Epidemiol..

[6] Shah PS, Balkhair T, Ohlsson A, Beyene J, Scott F, Frick C (2011). Intention to become pregnant and low birth weight and preterm birth: A systematic review. Matern. Child Health J..

[7] Russell RB (2007). Cost of hospitalization for preterm and low birth weight infants in the United States. Pediatrics.

[8] Windham GC, Hopkins B, Fenster L, Swan SH (2000). Prenatal active or passive tobacco smoke exposure and the risk of preterm delivery or low birth weight. Epidemiology.

[9] Rahman MO (2023). Detecting geographical clusters of low birth weight and/or preterm birth in Japan. Sci. Rep..

[10] Grote NK, Bridge JA, Gavin AR, Melville JL, Iyengar S, Katon WJ (2010). A meta-analysis of depression during pregnancy and the risk of preterm birth, low birth weight, and intrauterine growth restriction. Arch. Gen. Psychiatry.

[11] Stieb DM, Chen L, Eshoul M, Judek S (2012). Ambient air pollution, birth weight and preterm birth: A systematic review and meta-analysis. Environ. Res..

[12] Taha Z, Hassan AA, Wikkeling-Scott L, Papandreou D (2020). Factors associated with preterm birth and low birth weight in Abu Dhabi, the United Arab Emirates. Int. J. Environ. Res. Public Health.

[13] Desiani, A., Primartha, R., Arhami, M. & Orsalan, O. Naive bayes classifier for infant weight prediction of hypertension mother. In *Journal of Physics: Conference Series*, 012005 (Institute of Physics Publishing, 2019). 10.1088/1742-6596/1282/1/012005

[14] Reduction of Low Birth Weight: A South Asia Priority—PDF Free Download. Accessed 11 Jan 2021. [Online]. Available: https://docplayer.net/20755175-Reduction-of-low-birth-weight-a-south-asia-priority.html

[15] Li J (2020). Comparison of different machine learning approaches to predict small for gestational age infants. IEEE Trans. Big Data.

[16] Liu L (2016). Global, regional, and national causes of under-5 mortality in 2000–15: An updated systematic analysis with implications for the sustainable development goals. Lancet.

[17] Blencowe H (2013). Born too soon: The global epidemiology of 15 million preterm births. Reprod. Health.

[18] Lincetto O, Banerjee A (2020). World prematurity day: Improving survival and quality of life for millions of babies born preterm around the world. Am. J. Physiol.-Lung Cell. Mol. Physiol..

[19] Zimmermann LJI, Kostenzer J, Mader S (2020). Tackling bronchopulmonary dysplasia to improve preterm health: A call for family-centered care at World Prematurity Day 2020. Am. J. Physiol.-Lung Cell. Mol. Physiol..

[20] Preterm birth is associated with xenobiotics and predicted by the vaginal metabolome | Nature Microbiology. Accessed 08 Feb 2023. [Online]. Available: https://www.nature.com/articles/s41564-022-01293-8 10.1038/s41564-022-01293-8 PMC9894755 36635575

[21] Wu Z, Pan S, Chen F, Long G, Zhang C, Yu PS (2021). A comprehensive survey on graph neural networks. IEEE Trans. Neural Netw. Learn. Syst..

[22] Du X, Yu J, Chu Z, Jin L, Chen J (2022). Graph autoencoder-based unsupervised outlier detection. Inf. Sci..

[23] Feng M, Wan L, Li Z, Qing L, Qi X (2019). Fetal weight estimation via ultrasound using machine learning. IEEE Access.

[24] Campos Trujillo, O., Perez-Gonzalez, J. & Medina-Bañuelos, V. Early prediction of weight at birth using support vector regression. In *IFMBE Proceedings*, 37–41 (Springer, 2020). 10.1007/978-3-030-30648-9_5

[25] Khan W (2023). Infant low birth weight prediction using graph embedding features. Int. J. Environ. Res. Public Health.

[26] Khan W (2022). Infant birth weight estimation and low birth weight classification in United Arab Emirates using machine learning algorithms. Sci. Rep..

[27] Mercer BM (1996). The preterm prediction study: A clinical risk assessment system. Am. J. Obstet. Gynecol..

[28] Lee KS, Ahn KH (2019). Artificial neural network analysis of spontaneous preterm labor and birth and its major determinants. J. Korean Med. Sci..

[29] Tran, T., Luo, W., Phung, D., Morris, J., Rickard, K. & Venkatesh, S. Preterm birth prediction: Deriving stable and interpretable rules from high dimensional data. 10.48550/arxiv.1607.08310 (2016)

[30] Sun Q (2022). Machine learning-based prediction model of preterm birth using electronic health record. J. Healthc. Eng..

[31] Koivu A, Sairanen M (2020). Predicting risk of stillbirth and preterm pregnancies with machine learning. Health Inf. Sci. Syst..

[32] Kuhle S (2018). Comparison of logistic regression with machine learning methods for the prediction of fetal growth abnormalities: A retrospective cohort study. BMC Pregnancy Childbirth.

[33] Belaghi RA, Beyene J, McDonald SD (2021). Prediction of preterm birth in nulliparous women using logistic regression and machine learning. PLOS ONE.

[34] Borson, N. S., Kabir, M. R., Zamal, Z. & Rahman, R. M. Correlation analysis of demographic factors on low birth weight and prediction modeling using machine learning techniques. In *Proceedings of the World Conference on Smart Trends in Systems, Security and Sustainability, WS4 2020*, 169–173 (Institute of Electrical and Electronics Engineers Inc., 2020). 10.1109/WorldS450073.2020.9210338

[35] Loreto, P., Peixoto, H., Abelha, A. & Machado, J. Predicting low birth weight babies through data mining. In *Advances in Intelligent Systems and Computing*, 568–577 (Springer Verlag, 2019). 10.1007/978-3-030-16187-3_55

[36] Arabi Belaghi R, Beyene J, McDonald SD (2021). Clinical risk models for preterm birth less than 28 weeks and less than 32 weeks of gestation using a large retrospective cohort. J. Perinatol..

[37] Díaz E, Fernández-Plaza C, Abad I, Alonso A, González C, Díaz I (2021). Machine learning as a tool to study the influence of chronodisruption in preterm births. J. Ambient Intell. Humaniz. Comput..

[38] Lee KS (2021). Association of preterm birth with depression and particulate matter: Machine learning analysis using national health insurance data. Diagnostics.

[39] Al Haddad A (2019). Mutaba’ah—Mother and Child Health Study: Protocol for a prospective cohort study investigating the maternal and early life determinants of infant, child, adolescent and maternal health in the United Arab Emirates. BMJ Open.

[40] Ma X (2021). A comprehensive survey on graph anomaly detection with deep learning. IEEE Trans. Knowl. Data Eng..

[41] Tsuang M (2000). Schizophrenia: Genes and environment. Biol. Psychiatry.

[42] Grover, A. & Leskovec, J. node2vec: Scalable feature learning for networks. In *Proceedings of the 22nd ACM SIGKDD International Conference on Knowledge Discovery and Data Mining*, in KDD ’16. 855–864 (Association for Computing Machinery, New York, NY, USA, 2016). 10.1145/2939672.2939754 10.1145/2939672.2939754 PMC5108654 27853626

[43] Chen, H., Sultan, S. F., Tian, Y., Chen, M. & Skiena, S. Fast and accurate network embeddings via very sparse random projection. arXiv, Aug 29, 2019. Accessed Mar 11 2023. [Online]. Available: http://arxiv.org/abs/1908.11512

[44] Davis, J. & Goadrich, M. The relationship between precision-recall and ROC curves. In *Proceedings of the 23rd International Conference on Machine Learning—ICML ’06*, 233–240 (ACM Press, Pittsburgh, Pennsylvania, 2006). 10.1145/1143844.1143874

[45] Rose MS, Pana G, Premji S (2016). Prenatal maternal anxiety as a risk factor for preterm birth and the effects of heterogeneity on this relationship: a systematic review and meta-analysis. Biomed. Res. Int..

[46] Romero R, Espinoza J, Gonçalves LF, Kusanovic JP, Friel L, Hassan S (2007). The role of inflammation and infection in preterm birth. Semin. Reprod. Med..

[47] Ion R, Bernal AL (2015). Smoking and preterm birth. Reprod. Sci..

[48] Choltus H (2021). Cigarette smoke condensate exposure induces receptor for advanced glycation end-products (RAGE)-dependent sterile inflammation in amniotic epithelial cells. Int. J. Mol. Sci..

[49] Senthilkumar, D. & Paulraj, S, Prediction of Low Birth Weight Infants and Its Risk Factors Using Data Mining Techniques.

[50] Kumar SN (2020). Predicting risk of low birth weight offspring from maternal features and blood polycyclic aromatic hydrocarbon concentration. Reprod. Toxicol..

[51] Yarlapati, A. R., Roy Dey, S. & Saha, S. Early prediction of LBW cases via minimum error rate classifier: A statistical machine learning approach. In *2017 IEEE International Conference on Smart Computing, SMARTCOMP 2017*, (Institute of Electrical and Electronics Engineers Inc., 2017). 10.1109/SMARTCOMP.2017.7947002

[52] Faruk A, Cahyono ES, Eliyati N, Arifieni I (2018). Prediction and classification of low birth weight data using machine learning techniques. Indones. J. Sci. Technol..

[53] Akhtar F (2019). Diagnosis and prediction of large-for-gestational-age fetus using the stacked generalizationmethod. Appl. Sci..

[54] Akhtar F (2020). Effective large for gestational age prediction using machine learning techniques with monitoring biochemical indicators. J. Supercomput..

[55] Al Habashneh R, Khader YS, Al Jabali O, Alchalabi H (2013). Prediction of preterm and low birth weight delivery by maternal periodontal parameters: Receiver operating characteristic (ROC) curve analysis. Matern. Child Health J..

[56] Ahmadi P (2017). Prediction of low birth weight using random forest: A comparison with logistic regression. J. Paramed. Sci..

[57] Hussain Z, Borah MD (2020). Birth weight prediction of new born baby with application of machine learning techniques on features of mother. J. Stat. Manag. Syst..

[58] Lu, Y., Zhang, X., Fu, X., Chen, F. & Wong, K. K. L. Ensemble machine learning for estimating fetal weight at varying gestational age. In *33rd AAAI Conference on Artificial Intelligence, AAAI 2019, 31st Innovative Applications of Artificial Intelligence Conference, IAAI 2019 and the 9th AAAI Symposium on Educational Advances in Artificial Intelligence, EAAI 2019*, 9522–9527 (AAAI Press, 2019). 10.1609/aaai.v33i01.33019522

[59] Akbulut A, Ertugrul E, Topcu V (2018). Fetal health status prediction based on maternal clinical history using machine learning techniques. Comput. Methods Programs Biomed..

[60] Raja R, Mukherjee I, Sarkar BK (2021). A machine learning-based prediction model for preterm birth in Rural India. J. Healthc. Eng..

